# Mr. Shaw

**Published:** 1827-08

**Authors:** 


### OBITUARY.

It is with sentiments of no common regret that we have to announce the death of Mr. Shaw, surgeon to the Middlesex Hospital, which took place on the 19th ult. in the thirty-sixth year of his age. He was attacked with fever towards the end of May, which soon assumed a formidable character, and ran a very protracted course: at length, however, he became so far convalescent as to admit of his being moved into the country, where he continued to improve, and the most sanguine expectations were entertained of his ultimate recovery. On the 17th, however, he experienced a sudden relapse, and survived only two days.

Mr. Shaw was for many years demonstrator of anatomy in the School of Great Windmill-street, where his constant attention to their interests, and the frank and unreserved manner in which he entered into their views and feelings, made him greatly beloved by the pupils, among whom he formed many personal friendships, which continued in after-life. On the death of Mr. Wilson, he succeeded him as joint lecturer with Mr. C. Bell; and, on the resignation of Mr. Cartwright, he was elected one of the surgeons to the Middlesex Hospital.

Few men of Mr. Shaw's standing in the profession have been more assiduous in its cultivation: his Manual of Anatomy, his various works on the Spine, and numerous papers on the Nerves and other subjects, are sufficient proofs of his industry and his talents. His attention during the last few years was particularly directed to the subject of spinal distortions; and, while his investigations have thrown much additional light upon the pathology and treatment of these complaints, they at the same time had acquired for him a lucrative and rapidly increasing business.

Mr. Shaw was a man of thoroughly honourable mind; a circumstance which led him to express himself strongly at the appearance of any thing unfair in others: in disposition he was single-hearted; in manners open and unreserved to an extent not often met with. Such, for we *knew* him, was the character of Mr. Shaw. That he should have been cut off in the prime of his life, and, after twenty years of toil, just when he had begun to reap the fruits of labour, is a striking illustration of the precarious tenure of our condition, and the fallacy of our most rational hopes.

In concluding this short tribute to his memory, we may say that no one who knew him will hear of his early death without feeling that the profession has lost a useful and meritorious member, and society a man of amiable disposition and unblemished integrity.

